# Pomegranate Extract (POMx) Induces Mitochondrial Dysfunction and Apoptosis of Oral Cancer Cells

**DOI:** 10.3390/antiox10071117

**Published:** 2021-07-13

**Authors:** Sheng-Yao Peng, Li-Ching Lin, Shu-Rong Chen, Ammad A. Farooqi, Yuan-Bin Cheng, Jen-Yang Tang, Hsueh-Wei Chang

**Affiliations:** 1 Department of Biomedical Science and Environmental Biology, Ph.D Program in Life Sciences, College of Life Sciences, Kaohsiung Medical University, Kaohsiung 80708, Taiwan; u109851101@kmu.edu.tw; 2 Department of Radiation Oncology, Chi-Mei Foundation Medical Center, Tainan 71004, Taiwan; 8508a6@mail.chimei.org.tw; 3 School of Medicine, Taipei Medical University, Taipei 11031, Taiwan; 4 Chung Hwa University Medical Technology, Tainan 71703, Taiwan; 5 Graduate Institute of Natural Products, Kaohsiung Medical University, Kaohsiung 80708, Taiwan; u106831002@kmu.edu.tw; 6 Institute of Biomedical and Genetic Engineering (IBGE), Islamabad 54000, Pakistan; farooqiammadahmad@gmail.com; 7 Department of Marine Biotechnology and Resources, National Sun Yat-sen University, Kaohsiung 80424, Taiwan; jmb@mail.nsysu.edu.tw; 8 School of Post-Baccalaureate Medicine, Kaohsiung Medical University, Kaohsiung 80708, Taiwan; 9 Department of Radiation Oncology, Kaohsiung Medical University Hospital, Kaohsiung 80708, Taiwan; 10 Institute of Medical Science and Technology, National Sun Yat-sen University, Kaohsiung 80424, Taiwan; 11 Center for Cancer Research, Kaohsiung Medical University, Kaohsiung 80708, Taiwan

**Keywords:** pomegranate, mitochondrial DNA, DNA damage, apoptosis, oral cancer

## Abstract

The anticancer effect of pomegranate polyphenolic extract POMx in oral cancer cells has rarely been explored, especially where its impact on mitochondrial functioning is concerned. Here, we attempt to evaluate the proliferation modulating function and mechanism of POMx against human oral cancer (Ca9-22, HSC-3, and OC-2) cells. POMx induced ATP depletion, subG1 accumulation, and annexin V/Western blotting-detected apoptosis in these three oral cancer cell lines but showed no toxicity to normal oral cell lines (HGF-1). POMx triggered mitochondrial membrane potential (MitoMP) disruption and mitochondrial superoxide (MitoSOX) generation associated with the differential downregulation of several antioxidant gene mRNA/protein expressions in oral cancer cells. POMx downregulated mitochondrial mass, mitochondrial DNA copy number, and mitochondrial biogenesis gene mRNA/protein expression in oral cancer cells. Moreover, POMx induced both PCR-based mitochondrial DNA damage and γH2AX-detected nuclear DNA damage in oral cancer cells. In conclusion, POMx provides antiproliferation and apoptosis of oral cancer cells through mechanisms of mitochondrial impairment.

## 1. Introduction

Pomegranate has gained extraordinary appreciation because of its ability to inhibit/prevent a wide variety of cancers [1,2,3]. Pomegranate (*Punica granatum* L.) fruits contain abundant polyphenols [4,5]. The knowledge base in fields such as nutrigenetics and nutrigenomics continuously expands at a rapid rate. Emerging scientific evidence enables us to obtain a better understanding of the significant pharmacological properties of bioactive constituents derived from plants such as pomegranate [6]. Pomegranate, due to its bioactive compounds, belongs to a group of functional foods [7]. Its commercial dietary extract, POMx, is standardized with polyphenolic ellagitannin content, and its food safety is regarded as “generally recognized as being safe (GRAS)” by the U.S. Food and Drug Administration (FDA) [8]. POMx has several cellular and clinically relevant functions on several types of cancer [9,10,11,12,13].

Since pomegranate is an antioxidant-rich natural product, POMx may have similar effects. Antioxidants commonly have a dual function for reducing or inducing cellular oxidative stress, coming along with low or high doses [14]. For example, low-dose POMx (2.5 to 40 μg/mL) suppresses UVB-induced oxidative stress in keratinocyte HaCaT cells. In contrast, high dose POMx (100 to 200 μg/mL) may trigger oxidative stress in several types of cancer cell lines such as lung cancer, leukemia, and fibrosarcoma [15,16,17].

The oxidative stress function in cancer cell lines after POMx incubation has rarely been investigated [15,16,17]. A detailed examination in oral cancer cells for the response and mechanism to POMx is warranted. The antiproliferation ability of POMx against oral cancer cells has rarely been investigated as well. Recently, we reported that low cytotoxic doses of POMx suppressed transwell migration ability and Matrigel invasion behavior of human oral cancer cells [18]. However, the antioral cancer function at high-dose POMx remains unclear.

Mitochondria serve as the powerhouse of the cells and are responsible for the central source of oxidative stress, which regulates cellular energy supplies, proliferation, and apoptosis [19,20]. Although POMx was reported to induce apoptosis in several types of cancer cell lines [10,11,21,22], only mitochondrial apoptosis signaling such as caspases were studied. Other examinations for evaluating mitochondrial function such as mitochondrial membrane potential (MitoMP), mitochondrial superoxide (MitoSOX), mitochondrial mass, mitochondrial DNA (mtDNA) copy number, mtDNA lesion, and mitochondrial biogenesis were rarely investigated.

We aimed to test the hypothesis that oxidative stress generated by POMx provides apoptosis resulting in antiproliferation against oral cancer cells via mitochondrial impairment. Therefore, we evaluated ATP content, apoptosis, mitochondrial function, and DNA damage in the example of human oral cancer cells following POMx incubation.

## 2. Materials and Methods

### 2.1. Cell Culture and Drug Source

One normal oral cell line, HGF-1 (human normal gingival fibroblast), and two oral cancer cell lines (HSC-3 and Ca9-22) were commercially available, and one oral cancer cell line (OC-2) was provided by Dr. Wan-Chi Tsai (Kaohsiung Medical University, Kaohsiung, Taiwan) [23]. These cell lines were kept in culture medium with DMEM/F-12 (Dulbecco’s Modified Eagle Medium (DMEM)/Nutrient Mixture F-12) (Gibco, Grand Island, NY, USA) at a ratio of 3 vs. 2, containing penicillin, streptomycin, and 10% fetal bovine serum (Gibco).

POMx is a commercial pomegranate (*Punica granatum* L.)-derived polyphenols-rich aqueous extract powder (POM Wonderful, LLC, Los Angeles, CA, USA) [13,24]. The detailed characterization of chemicals in this POMx powder extract had been previously reported [9,24], such as ellagitannins (punicalagin and punicalin) and ellagic acid. POMx was immediately prepared in dimethyl sulfoxide (DMSO) before experiments.

### 2.2. Determination of Main Components of POMx by HPLC

Qualitative and quantitative analysis was performed on a Shimadzu HPLC (Kyoto, Japan) system, equipped with an LC-20AT prominence liquid chromatography, a SIL-40AD autosampler, and an SPD-M20A diode array detector. The determination was carried out with a reversed-phase column (Luna C_18_ column, 250 mm × 4.6 mm, 5 μm; Phenomenex; Torrance, CA, USA). The mobile phase consisted of (A) water with 0.1% (*v*/*v*) trifluoroacetic acid and (B) methanol. The gradient elution system was set as follows: 0−5 min, 1% B; 5−10 min 1−15% B; 10−15 min, 15% B; 15−35 min, 15−45% B; 35−40 min, 45−80% B; 40−45 min, 80−100% B; 45−50 min, 100% B. The flow rate was 1.0 mL/min. The injection was 10 μL, and the detection wavelength was 378 nm. The stock solutions were prepared by dissolving 1.0 mg of punicalin, punicalagin (Molnova; Ann Arbor, MI, USA), ellagic acid (Sigma-Aldrich; St. Louis, MO, USA), and POMx in 1.0 mL methanol. Six concentrations of three standards were prepared by diluting with methanol.

### 2.3. Cell Viability and Morphology

Viability was analyzed by an intracellular ATP content assay (PerkinElmer Life Sciences, Boston, MA, USA) [25] and trypan blue assay [26]. Cell morphology was observed at 100x magnification.

### 2.4. Cell Cycle Analysis

Cells were incubated with Biotium 7-aminoactinomycin D (7AAD) (Hayward, CA, USA) at the requirement of 1 μg/mL, 30 min, 37 °C, and DNA content was analyzed by Accuri C6 flow cytometer using FL3 channel (Becton-Dickinson, Mansfield, MA, USA) [27].

### 2.5. Annexin V/7AAD for Apoptosis Analysis

Cells were stained with annexin V mixed 7AAD kit (Strong Biotech Corp., Taipei, Taiwan) according to the user’s instructions, and signals analyzed by Accuri C6 flow cytometer (Becton-Dickinson) using FL1/FL3 channels as previously described [28].

### 2.6. Acridine Orange (AO) Staining for Autophagy Analysis

AO staining was used to detect acidic vesicular organelles as the fast screening for autophagy [29]. Cells were stained by 10 ng/mL AO (Sigma, St Louis, MO, USA) at the requirement (37 °C, 30 min) and analyzed by Accuri C6 flow cytometer (Becton-Dickinson) using FL3 channel as previously described [30].

### 2.7. Mitochondrial Membrane Potential (MitoMP)

Cells were stained by 5 nM MitoProbe^TM^ DiOC_2_(3) (Thermo Fisher Scientific, Carlsbad, CA, USA) at the requirement (37 °C, 30 min) and analyzed by Accuri C6 flow cytometer (Becton-Dickinson) using FL1 channel as previously described [31].

### 2.8. Mitochondrial Superoxide (MitoSOX) Generation

Cells were stained by 50 nM MitoSOX™ Red (Thermo Fisher Scientific) at the requirement (37 °C, 30 min) and analyzed by Accuri C6 flow cytometer (Becton-Dickinson) using FL3 channel as previously described [32].

### 2.9. Quantitative RT-PCR (qRT-PCR) Analysis: Antioxidant- and Mitochondrial Biogenesis-Related Genes

RNA was extracted and reverse-transcribed [33] to cDNA for qRT-PCR as described [34]. In addition, for antioxidant-related genes [35,36], nuclear factor erythroid 2-like 2 (*NFE2L2*), glutamate-cysteine ligase catalytic subunit (*GCLC*), thioredoxin (*TXN*), catalase (*CAT*), superoxide dismutase 1 (*SOD1*) [37], heme oxygenase 1 (*HMOX1*), and quinone dehydrogenase 1 (*NQO1*) were selected.

For mitochondrial biogenesis-related genes [38], transcription factor B2, mitochondrial (*TFB2M*), transcription factor A, mitochondrial (*TFAM*), RNA polymerase mitochondrial (*POLRMT*), and Tu translation elongation factor, mitochondrial (*TUFM*) [39] were selected. Touch-down PCR program (running for 50 cycles) [34] was applied to qRT-PCR reactions.

The qRT-PCR primer information for antioxidant- and mitochondrial biogenesis-related genes, as well as GAPDH, are provided in the top and bottom of Table 1, respectively. In reference to housekeeping gene *GAPDH*, the relative mRNA expression (log_2_) of these genes was calculated according to the 2^−ΔΔCt^ method [40]. In brief, ΔCt is calculated as (Ct value of a target gene—Ct value of *GAPDH* gene), where the target genes are antioxidant- and mitochondrial biogenesis-related genes. When the Ct value of a target gene is undetectable (>50 cycles; qRT-PCR is performed for 50 cycles), it was assigned 50 cycles for further calculation. The ΔΔCt is the difference in ΔCt between the drug treatment and untreated control, which is ΔΔCt = ΔCt (drug treatment) − ΔCt (control).

### 2.10. Mitochondrial Mass

For mitochondrial mass measurement, cells were stained by 300 nM MitoTracker^TM^ Green FM (Thermo Fisher Scientific) at the requirement (37 °C, 30 min) and analyzed by Accuri C6 flow cytometer (Becton-Dickinson) using FL1 channel as described [43].

### 2.11. Quantitative PCR (qPCR): mtDNA Copy Number

Total genomic DNA from cells incubated with POMx for 24 and 72 h was prepared according to the OMEGA Bio-Tek user manual of the E.Z.N.A.^®^ Tissue DNA kit (Norcross, GA, USA) [44]. Using the nuclear DNA (nDNA) gene *GAPDH* as a reference, the relative copy numbers of mtDNA such as NADH-ubiquinone oxidoreductase chain 1 (*ND1*) and *ND5* genes [45] were analyzed using the 2^−ΔΔCt^ method [40] after qPCR reaction in a touch-down program [34]. The PCR information for the mtDNA copy number is listed in Table 2.

### 2.12. Semi-Long Run Quantitative PCR (SLR-qPCR): mtDNA Damage

SLR-qPCR was applied to assess mtDNA damages [46]. Using SLR-qPCR, the copy numbers of two DNA fragments with different lengths, i.e., small (*ND1*/*ND5*) and long (*ND1*-L/*ND5*-L) fragments, were measured for calculating mtDNA damage (lesions per 10 kb DNA between *ND1* and *ND5*) by the formula: (1–2^−Δ (long Ct-short Ct)^) × 10,000 (bp)/length of the long fragment (bp) [46]. The primer and PCR amplicon information for mitochondrial DNA damage is provided in Table 2.

### 2.13. DNA Damage: γH2AX

The level of double-strand break marker for DNA damage (γH2AX) was analyzed [47]. p-Histone H2A.X (Ser 139) at 500X dilution was chosen as the primary antibody purchased from Santa Cruz Biotechnology (Santa Cruz, CA, USA) to detect γH2AX at 4 °C for 1 h. Subsequently, a secondary antibody conjugated by Alexa 488 was used in its flow cytometry application (BD Accuri C6; FL1 channel).

### 2.14. Western Blotting Analysis for Apoptosis, Antioxidant Signaling, Mitochondrial Resident Proteins, and Mitochondrial Biogenesis

All Western blotting routine steps were mentioned previously [48]. Apoptosis antibodies included cleaved poly (ADP-ribose) polymerase (c-PARP), Bcl-xL, Bcl-2, and Bax (Cell signaling; Danvers, MA, USA). Antioxidant signaling antibodies included nuclear factor erythroid 2-related factor 2 (NRF2) (Fine Test; Wuhan, China), catalase (Merck; Darmstadt, Germany), peroxiredoxin 1 (PRX1) (GeneTex; Irvine, CA, USA), and superoxide dismutase 1 (SOD1) (Abcam; Cambridge, UK). Mitochondrial resident protein antibodies included translocase of the inner membrane (TIMM22) (Proteintech; Rosemont, IL, USA) and translocase of outer mitochondrial membrane 20 (TOMM20) (Cell signaling). Mitochondrial biogenesis antibodies (Biorbyt; Cambridge, UK) included RNA polymerase mitochondrial (POLRMT), Tu translation elongation factor, mitochondrial (TUFM), transcription factor B2, mitochondrial (TFB2M), transcription factor A, and mitochondrial (TFAM). Except for antibodies against TFB2M (1:5000) and β-actin (Sigma-Aldrich; St. Louis, MO, USA) (1:10,000), all antibodies were used in 1:1000 dilution.

### 2.15. Statistics

One-way ANOVA processed all the statistics after Tukey’s HSD post hoc tests using JMP^®^12 software to compare different groups [49]. Treatments without overlapping low cases are regarded as significant differences.

## 3. Results

### 3.1. HPLC profile of POMx and Three Main Bioactive Components

**Figure 1:**
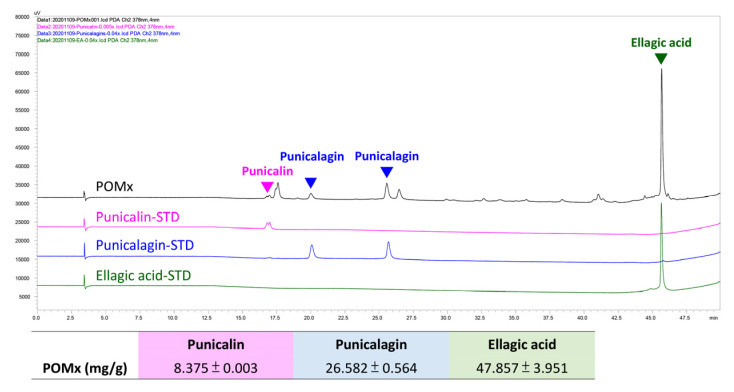
HPLC profile of POMx and the contents of its three main bioactive components. The HPLC profile for punicalagin, ellagic acid, and punicalin was provided as well as their contents within POMx (mg/g). STD means standard.

The contents for punicalin, punicalagin, and ellagic acid of POMx-capsules were analyzed by HPLC using authentic reference compounds. The linear equations of three main compounds were y = 10^7^x − 69,990 (R^2^ = 0.9997), y = 5 × 10^6^x − 47,955 (R^2^ = 0.9998), and y = 7 × 10^6^x − 21,969 (R^2^ = 0.9999), respectively. The results show that POMx contains punicalagin 26.582 mg/g, ellagic acid 47.857 mg/g, and punicalin 8.375 mg/g (Figure 1).

### 3.2. Antiproliferation of Oral Cancer Cells Following POMx Incubation

**Figure 2:**
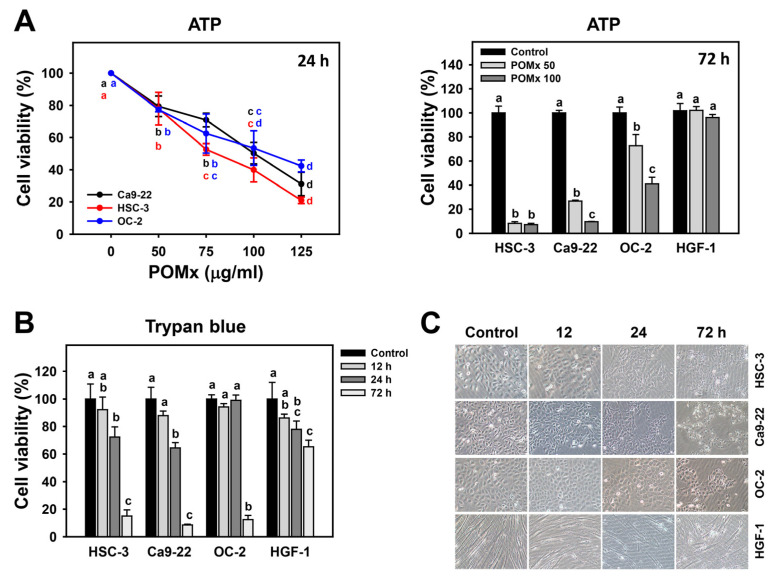
Cell viability and morphology of oral cancer cells after POMx incubation. Cells were incubated with 0, 50, and 100 μg/mL of POMx (control, POMx 50, and POMx 100) or other indicated concentrations for 24 or 72 h. (**A**) Cell viability for three oral cancer cell lines (HSC-3, Ca9-22, and OC-2) at 24 h and 72 h ATP assays. (**B**) Cell viability for three oral cancer cell lines (HSC-3, Ca9-22, and OC-2), and a normal oral cell line (HGF-1) at 0, 12, 24, and 72 h trypan blue assays. (**C**) Morphology for oral cancer cells and normal oral cells at 0, 12, 24, and 72 h for 100 μg/mL POMx incubation. Treatments without overlapping low cases (a to d) are significant differences for the same cell lines. *p* < 0.05. Data, mean ± SD (*n* = 3). The morphology image was photographed at 100× magnification.

Cell viability detected by ATP assay in oral cancer cells after POMx (0, 50, 75, 100, and 125 μg/mL) treatment for 24 h is dose-responsively decreased (Figure 2A). IC_50_ value at 24 h ATP assay for POMx in oral cancer cells (Ca9-22, HSC-3, and OC-2) are 80.53, 100.34, and 108.12 μg/mL, respectively. Moreover, longer exposure to POMx for 72 h decreases more viability to oral cancer cells than that of the 24 h treatment. In contrast, normal oral cells (HGF-1) show only a mild decrease after 72 h exposure to POMx.

Similarly, cell viability detected by trypan blue assay in oral cancer and normal oral cells after POMx (0 and 100 μg/mL) treatment for 0, 12, 24, and 72 h are time-dependently decreased (Figure 2B). In addition, it was noted that cell viabilities for oral cancer cells (Ca9-22, HSC-3, and OC-2) are lower than that of normal oral cells.

Figure 2C shows that 24 and 72 h POMx incubations of oral cancer cells induce abnormal cell morphology while normal oral cells (HGF-1) retain normal morphology. Accordingly, POMx has a selective killing effect on oral cancer cells but less harmful to normal oral cells.

### 3.3. Cell Cycle Change of Oral Cancer Cells Following POMx Incubation

**Figure 3:**
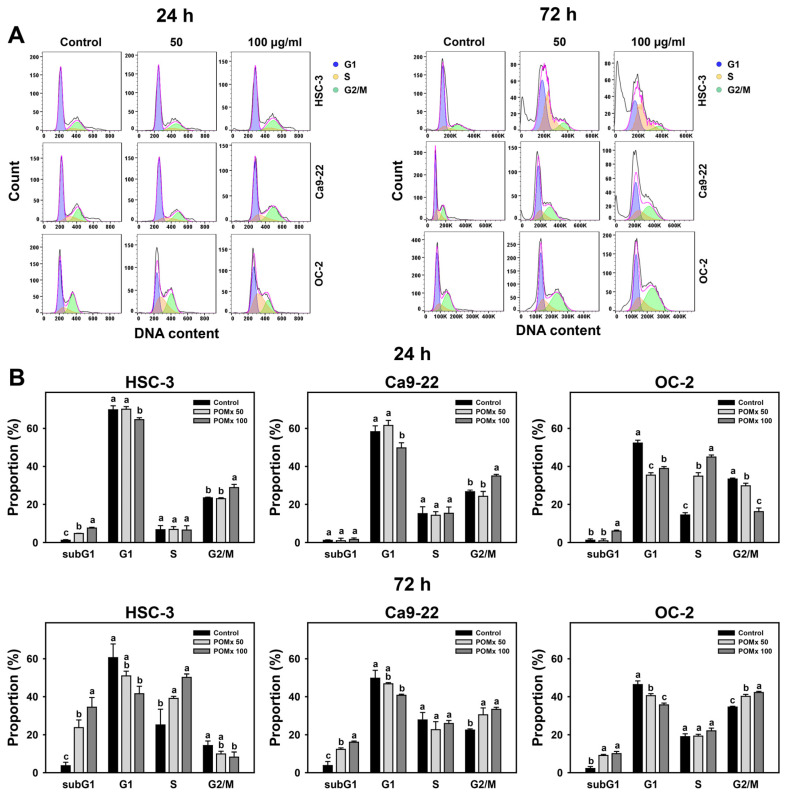
Cell cycle phase of oral cancer cells after POMx incubation. (**A**) Flow cytometry patterns. Cells (HSC-3, Ca9-22, and OC-2) were incubated with control, 50, and 100 μg/mL of POMx (control, POMx 50, and POMx 100) for 24 and 72 h. (**B**) Statistics for (**A**). Treatments without overlapping low cases (a to c) represent significant differences for the same cell lines. *p* < 0.05. Data, mean ± SD (*n* = 3).

After POMx incubations (0, 50, and 100 μg/mL) for 24 and 72 h, the patterns for cell cycles in three oral cancer cell lines are shown (Figure 3A). For 24 h POMx incubation, HSC-3 and OC-2 cells show slightly sub-G1 accumulations but not for Ca9-22 (Figure 3B). For 100 μg/mL POMx incubation, all these three cell lines show a decrease in the G1 phase. HSC-3 and Ca9-22 cells show an increase to G2/M, while OC-2 cells show an increase to S phase and G2/M decrease.

For 72 h POMx incubation, HSC-3 cells show dramatic sub-G1 and S phase accumulations but show a decrease in G1 and G2/M phases (Figure 3B). Ca9-22 and OC-2 cells show moderate subG1 and G2/M accumulation but show decreased G1 phase compared with the control.

Accordingly, POMx differentially disturbs cell cycle distribution of oral cancer cells between 24 and 72 h, and POMx at 72 h induces more subG1 accumulation (apoptosis-like) than at 24 h.

### 3.4. Apoptosis and Autophagy Changes of Oral Cancer Cells Following POMx Incubation

**Figure 4:**
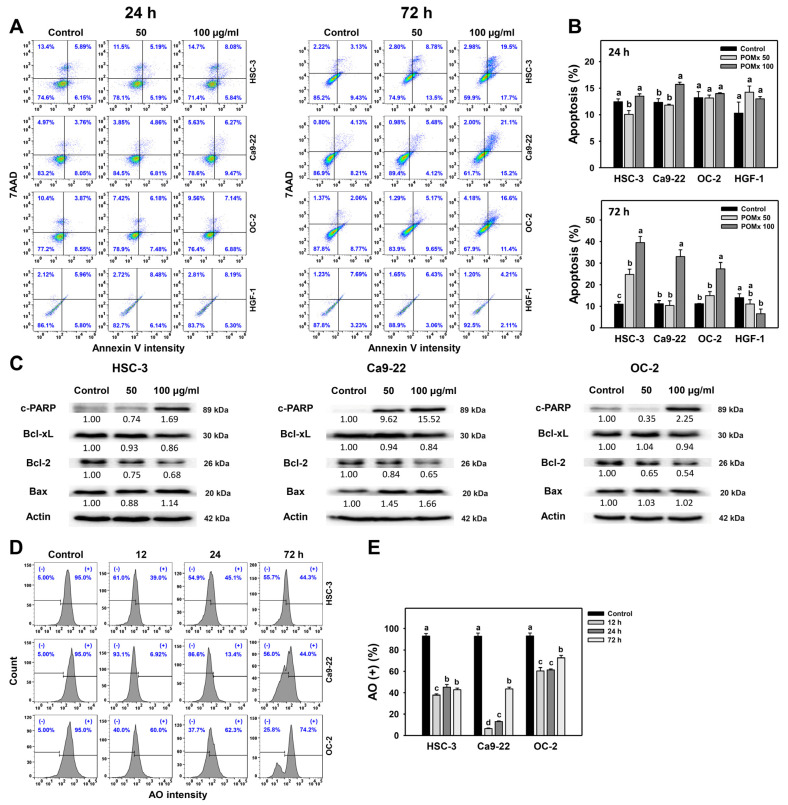
Apoptosis and autophagy changes of oral cancer cells after POMx incubation. Cells (HSC-3, Ca9-22, and OC-2) were incubated with control, 50, and 100 μg/mL of POMx (control, POMx 50, and POMx 100) for 24 and 72 h. (**A**) Annexin V/7AAD flow cytometry patterns. (**B**) Statistics for (**A**). (**C**) Western blotting for apoptosis marker expressions after 72 h POMx (0, 50, and 100 μg/mL) incubation. (**D**) Acridine orange (AO) flow cytometry patterns at 0, 12, 24, and 72 h for 100 μg/mL POMx incubation (**E**) Statistics for (**D**). Treatments without overlapping low cases (a to d) are significant differences for the same cell type. *p* < 0.001. Data, mean ± SD (*n* = 3).

After POMx incubations (0, 50, and 100 μg/mL) for 24 and 72 h, the dual staining patterns for annexin V/7AAD in oral cancer and normal cell lines are shown (Figure 4A). For 24 h POMx incubation, apoptosis (%) counting for annexin V (+)/7AAD (+ or −) population in oral cancer (HSC-3, Ca9-22, and OC-2) and normal oral cells (HGF-1) are weakly changed (Figure 4B).

In addition, POMx induces relatively more apoptosis in oral cancer cells than in normal oral cells. Moreover, apoptosis proteins such as cleaved PARP and BAX are increased, and the anti-apoptosis proteins such as Bcl-2 and Bcl-xL are decreased after 72 h POMx incubation (Figure 4C).

Moreover, the AO-detected autophagy of three oral cancer cell lines is decreased by POMx during 12, 24, and 72 h incubations compared with the control, suggesting that POMx may inter-regulate apoptosis and autophagy. Accordingly, 72 h POMx incubation induces more apoptosis than 24 h for oral cancer cells. Moreover, POMx induces more apoptosis in oral cancer cells than in normal oral cells, especially for 100 μg/mL at 72 h.

### 3.5. MitoMP of Oral Cancer Cells Following POMx Incubation

**Figure 5:**
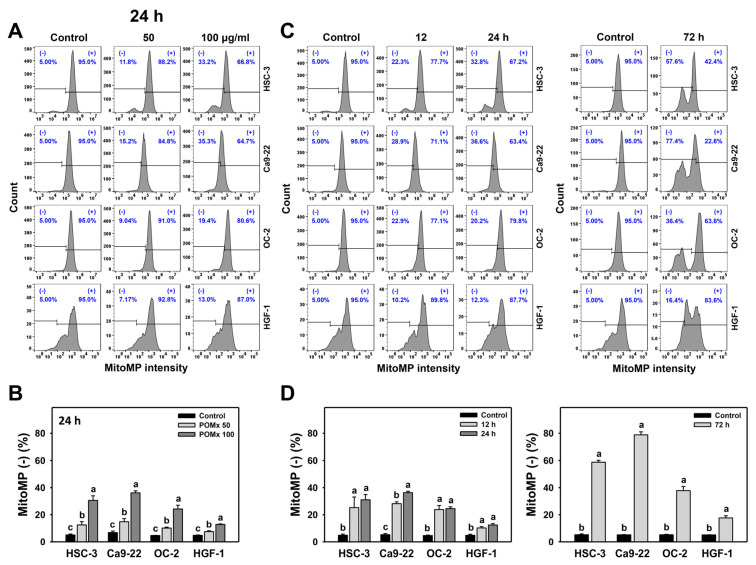
MitoMP change of oral cancer cells after POMx incubation. (**A**) Dose-response to MitoMP flow cytometry patterns. Cells (HSC-3, Ca9-22, and OC-2) were incubated with control, 50, and 100 μg/mL of POMx (control, POMx 50, and POMx 100) for 24 h. The MitoMP-negative (−) population was defined on the left part. (**B**) Statistics for (**A**). (**C**) Time course to MitoMP flow cytometry patterns. Cells were incubated with control and 100 μg/mL of POMx for 0, 12, 24, and 72 h. (**D**) Statistics for (**C**). Treatments without overlapping low cases (a to c) represent significant differences for the same cell lines. *p* < 0.01. Data, mean ± SD (*n* = 3).

After POMx incubation (0, 50, and 100 μg/mL) for 24 h, the patterns for MitoMP in oral cancer and normal (HGF-1) cell lines are shown (Figure 5A). The MitoMP (−) (%) of these three oral cancer cells dose-responsively increase after POMx incubation (Figure 5B). Moreover, POMx induces more MitoMP (−) (%) in three oral cancer cells than normal oral cells.

After time course treatments of POMx, the dynamics of flow cytometry patterns for MitoMP in these oral cancer cells are shown (Figure 5C). The MitoMP (−) (%) of these three oral cancer cells is increased over time (12, 24, and 72 h) after POMx incubation compared with the control (Figure 5D). Moreover, POMx induces more MitoMP (−) (%) in three oral cancer cells than in normal oral cells throughout the time course. Accordingly, POMx causes higher MitoMP destruction in oral cancer cells than in normal oral cells.

### 3.6. MitoSOX Generation of Oral Cancer Cells Following POMx Incubation

**Figure 6:**
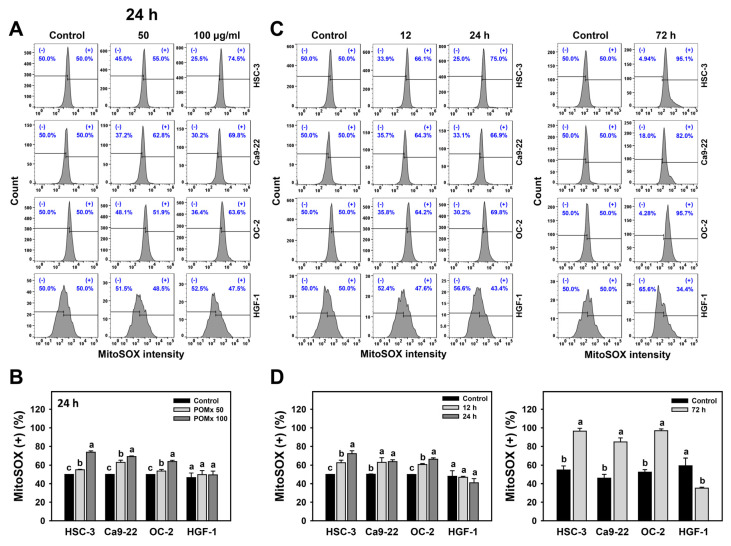
MitoSOX generation of oral cancer cells after POMx incubation. (**A**) Dose-response to MitoSOX flow cytometry patterns. Cells (HSC-3, Ca9-22, and OC-2) were incubated with 0, 50, and 100 μg/mL of POMx (control, POMx 50, and POMx 100) for 24 h. The MitoSOX (+) population was defined in the right part. (**B**) Statistics for (**A**). (**C**) Time course to MitoSOX flow cytometry patterns. Cells were incubated with control and 100 μg/mL of POMx for 0, 12, 24, and 72 h. (**D**) Statistics for (**C**). Treatments without overlapping low cases (a to c) show significant differences for the same cell lines. *p* < 0.05. Data, mean ± SD (*n* = 3).

After POMx incubations (0, 50, and 100 μg/mL) for 24 h, the patterns for MitoSOX in oral cancer (Ca9-22, HSC-3, and OC-2) and normal oral (HGF-1) cells are shown (Figure 6A). The MitoSOX (+) (%) of these three oral cancer cells were dose-responsively increased after POMx incubation while remaining unchanged in normal cells (Figure 6B).

After time course treatments of POMx, the flow cytometry patterns for MitoSOX in oral cancer and normal oral cells are shown (Figure 6C). The MitoSOX (+) (%) of these three oral cancer cells was increased over time (12, 24, and 72 h) after POMx incubation compared with the control, while it was unchanged in normal cells at 12 and 24 h and decreased at 72 h (Figure 6D). Accordingly, POMx induced higher MitoSOX generation in oral cancer cells than normal oral cells.

### 3.7. Antioxidant Gene Expression of Oral Cancer Cells Following POMx Incubation

**Figure 7:**
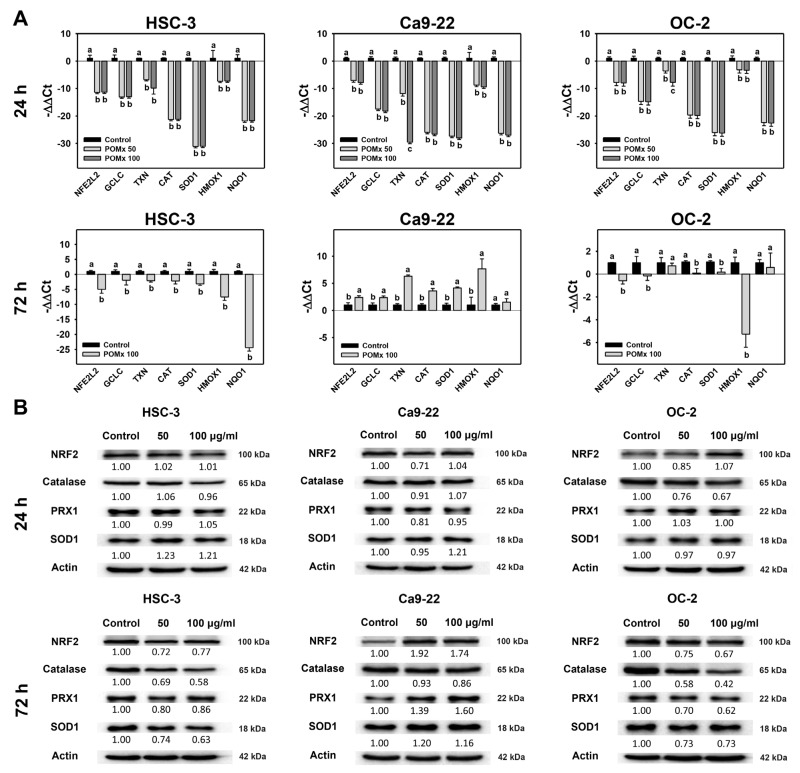
mRNA and protein expressions of antioxidant genes of oral cancer cells after POMx incubation. (**A**) Relative mRNA expressions (log_2_) of antioxidant genes of oral cancer cells after POMx incubation for 24 and 72 h. (**B**) Western blotting for antioxidant signaling proteins for 24 and 72 h. Cells were incubated with 0, 50, and 100 μg/mL of POMx (control, POMx 50, and POMx 100) for 24 and 72 h. Treatments without overlapping low cases (a to c) indicate significant differences for the same cell lines. *p* < 0.05. Data, mean ± SD (*n* = 3).

Inhibition of antioxidant pathways may induce increased oxidative stress [50]. Accordingly, the antioxidant signaling gene expression for mRNA [35], including *NFE2L2*, *GCLC*, *TXN*, *CAT*, *SOD1*, *HMOX1*, and *NQO1*, was examined for POMx-incubated oral cancer cells. After 24 h POMx incubations (0, 50, and 100 μg/mL), the relative mRNA gene expressions of these antioxidant genes in oral cancer (Ca9-22, HSC-3, and OC-2) cells were downregulated compared with the control (Figure 7A, top). However, for 24 h POMx incubations, the protein expressions of these antioxidant genes in oral cancer (Ca9-22, HSC-3, and OC-2) cells were almost unchanged (Figure 7B, top). Accordingly, mRNA and protein expressions were regulated differentially at 24 h POMx incubation.

For 72 h POMx incubations, the relative gene expressions of these antioxidant genes show differential expressions in these three oral cancer cell lines (Figure 7A, bottom). For example, 72 h POMx suppressed antioxidant mRNA gene expressions in HSC-3 and OC-2 cells, but these were induced in Ca9-22 cells. For 72 h POMx incubations, the protein expressions of these antioxidant genes were suppressed in HSC-3 and OC-2 cells but induced in Ca9-22 cells (Figure 7B, bottom). Accordingly, mRNA and protein expressions were consistently expressed at 72 h POMx incubation.

### 3.8. Mitochondrial Mass of Oral Cancer Cells Following POMx Incubation

**Figure 8:**
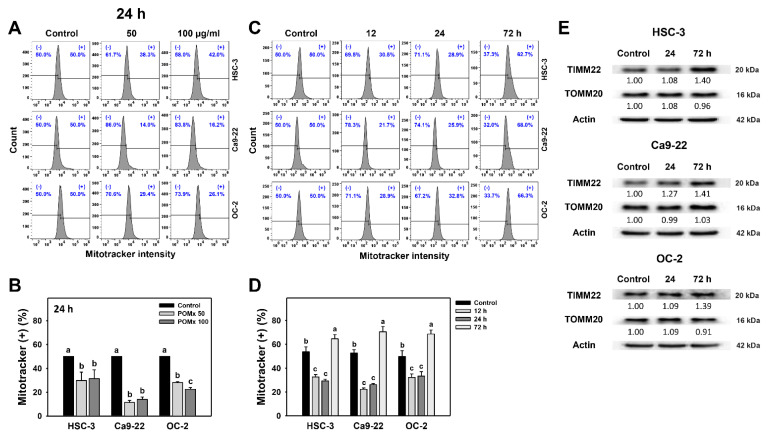
Mitochondrial mass of oral cancer cells after POMx incubation. (**A**) Dose-response to Mitotracker flow cytometry patterns. Cells (HSC-3, Ca9-22, and OC-2) were incubated with control, 50, and 100 μg/mL of POMx (control, POMx 50, and POMx 100) for 24 h. The Mitotracker (+) population was defined on the right side of each panel. (**B**) Statistics for (**A**). (**C**) Time course to Mitotracker flow cytometry patterns. Cells were incubated with control and 100 μg/mL of POMx for 0, 12, 24, and 72 h. (**D**) Statistics for (**C**). Treatments without overlapping low cases (a to c) are significantly different for the same cell lines. *p* < 0.05. Data, mean ± SD (*n* = 3). (**E**) Western blotting for mitochondrial resident proteins for 24 and 72 h. Cells were incubated with control and 100 μg/mL of POMx for 24 and 72 h.

After 24 h POMx incubation (0, 50, and 100 μg/mL), the patterns for Mitotracker in three oral cancer cell lines are shown (Figure 8A). The Mitotracker (+) (%) of these three oral cancer cells were decreased after POMx incubation compared with the control (Figure 8B).

After time course treatments of POMx, the flow cytometry patterns for Mitotracker in these oral cancer cells are shown (Figure 8C). The Mitotracker (+) (%) of these three oral cancer cells are decreased at 12 and 24 h POMx incubation compared with the control, although it was slightly increased at 72 h (Figure 8D).

After time course treatments of POMx, the mitochondrial resident protein (TIMM22 and TOMM20) expressions were detected in oral cancer cells (Figure 8E). For 24 h POMx incubations, the TIMM22 and TOMM20 are almost unchanged in oral cancer cells. However, although TOMM20 remains unchanged, TIMM22 is upregulated at 72 h POMx incubations, consistent with Mitotracker detection. Accordingly, Mitotracker detections and protein expressions are differentially regulated at 24 h POMx incubation but in a consistently regulated manner at 72 h.

### 3.9. Mitochondrial DNA Copy Number, Lesion and Biogenesis of Oral Cancer Cells Following POMx Incubation

**Figure 9:**
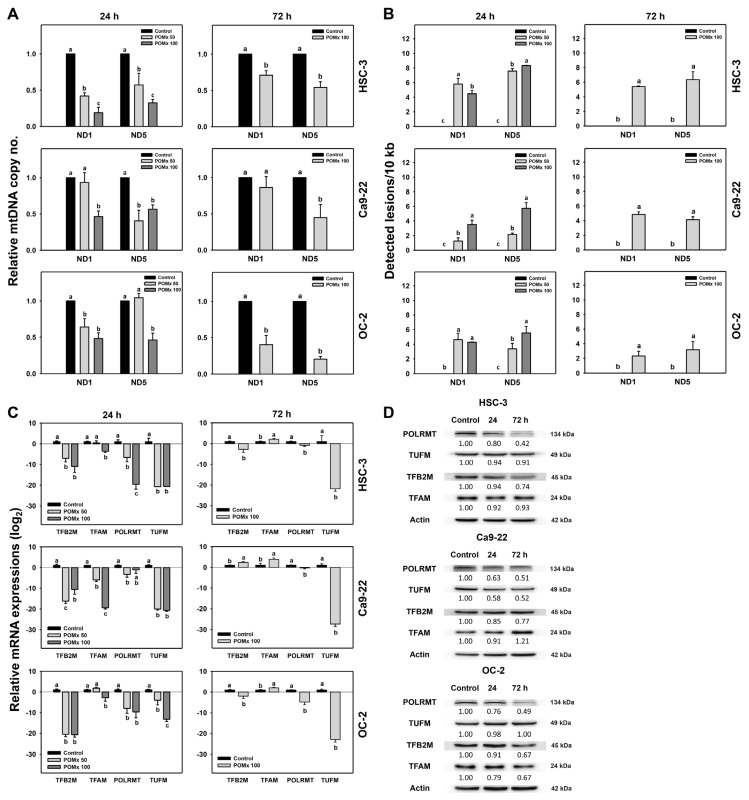
mtDNA copy number, mtDNA lesion, and mitochondrial biogenesis of oral cancer cells after POMx incubation. Cells (HSC-3, Ca9-22, and OC-2) were incubated with 0, 50, and 100 μg/mL of POMx (control, POMx 50, and POMx 100) for 24 and 72 h. (**A**) Relative mtDNA copy number in *ND1* and *ND5* genes. (**B**) mtDNA lesion frequency per 10 kb DNA between *ND1* to *ND5* genes. (**C**) Relative mRNA expressions (log_2_) for mitochondrial biogenesis genes. Treatments without overlapping low cases (a to c) are significantly different for the same cell lines. *p* < 0.05. Data, mean ± SD (*n* = 3). (**D**) Western blotting for mitochondrial biogenesis genes at 24 and 72 h POMx incubations. Cells were incubated with control and 100 μg/mL of POMx for 24 and 72 h.

In addition to MitoMP, MitoSOX, and mitochondrial mass as described above (Figure 5, Figure 6, Figure 7 and Figure 8), other mitochondrial functions such as mitochondrial DNA copy number, lesion, and biogenesis were further examined in POMx-incubated oral cancer cells (Figure 9). After POMx incubations (0, 50, and 100 μg/mL) for 24 and 72 h, the relative mtDNA copy numbers of three oral cancer cell lines were dose-responsively decreased (Figure 9A).

mtDNA damages between *ND1* and *ND5* genes were higher in oral cancer cells following 24 and 72 h POMx incubation than those of the control (Figure 9B). Moreover, the mRNA expressions of all tested mitochondrial biogenesis genes (*TFB2M*, *TFAM*, *POLRMT*, and *TUFM*) were downregulated by 24 h POMx compared with the control (Figure 9C, left). Among these biogenesis genes, TUFM was dramatically downregulated by 72 h POMx (Figure 9C, right). The protein expressions of these mitochondrial biogenesis genes were consistently downregulated at 24 and 72 h POMx (Figure 9D). Therefore, POMx downregulates gene expressions for mitochondrial biogenesis in oral cancer cells.

### 3.10. γH2AX-Detected DNA Damage of Oral Cancer Cells Following POMx Incubation

**Figure 10:**
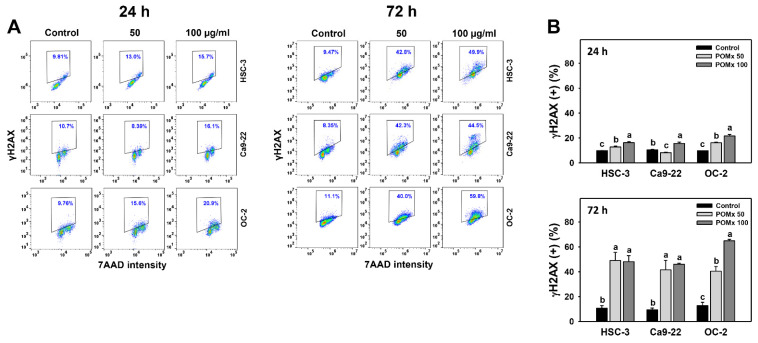
γH2AX change of oral cancer cells after POMx incubation. (**A**) Dose-response to γH2AX flow cytometry patterns. Cells (HSC-3, Ca9-22, and OC-2) were incubated with control, 50, and 100 μg/mL of POMx (control, POMx 50, and POMx 100) for 0, 24, and 72 h. The γH2AX (+) population (%) is marked within a dashed line. (**B**) Statistics of γH2AX (+) (%) in (**A**). Treatments without overlapping low cases (a to c) are significantly different for the same cell lines. *p* < 0.001. Data, mean ± SD (*n* = 3).

After POMx incubations (0, 50, and 100 μg/mL) for 0, 24, and 72 h, the patterns for γH2AX in three oral cancer cell lines were shown (Figure 10A). The γH2AX (+) (%) of these three oral cancer cells was slightly increased at 24 h POMx incubation and dramatically increased at 72 h POMx incubation compared with the control (Figure 10B).

Accordingly, POMx triggers γH2AX-detected DNA damage in oral cancer cells.

## 4. Discussion

We found that POMx showed antiproliferation, apoptosis, oxidative stress, mitochondrial impairment, and DNA damage to several kinds of oral cancer cells. The detailed mechanisms for the POMx-induced antiproliferation are discussed in the following.

### 4.1. POMx Has a Selective Antiproliferation Function towards Cancer Cells with Safety to Normal Cells

POMx provides antioxidant-rich natural products [51] and shows anticancer effects on several cancer cells [11,16,52]. This is partly explained by antioxidants having dual functions to reduce or induce oxidative stress at physiological or high concentrations [14]. The present study shows that the IC_50_ values at the 24 h ATP assay for POMx incubated three oral cancer cell lines (Ca9-22, HSC-3, and OC-2) were 80.53, 100.34, and 108.12 μg/mL, respectively (Figure 2). Similarly, IC_50_ values at 72 h MTS assay for POMx for prostate (C4-2, PC3, and ARCaPM) [11] were 42, 78, and 161 μg/mL, respectively. Moreover, trypan blue assay in addition to ATP assay confirms viability results of POMx in oral cancer and normal cells.

The safety of POMx is well documented. For example, normal human prostatic epithelial PrEC cells showed no cytotoxicity (95% viability) to POMx [11]. At the 72 h MTT assay, pomegranate fruit extract (PFE) (50–150 μg/mL) showed antiproliferation against lung cancer cells with 53% viability but no cytotoxic effects on normal bronchial epithelial cells with 90% viability [15]. The pomegranate juice and oil showed antiproliferation and apoptosis in prostate cancer cells but no cytotoxicity in normal prostate epithelial cells [53]. Similarly, the normal oral cells (HGF-1) show higher viability in both ATP assay and trypan blue assay (Figure 2A,B) than the three oral cancer cell lines of this study. Punicalagin and ellagic acid, two main components of POMx, induce apoptosis of colon cancer cells without affecting normal colon cells [54]. Therefore, POMx and other pomegranate-derived natural products provided selective killing against several cancer cells and did not show side effects on normal oral cells.

### 4.2. POMx Inhibits Antioxidant Signaling to Generate Oxidative Stress

When the pro-oxidant level is higher than the antioxidant level, cellular oxidative stress is generated. In addition, mitochondrial impairment may change antioxidant gene expressions [55]. For example, advanced glycation end products were reported to inhibit the cellular antioxidant system and trigger oxidative stress [50].

Similar to the present study, the mRNA expressions for several antioxidant genes (*NFE2L2*, *GCLC*, *TXN*, *CAT*, *SOD1*, *HMOX1*, and *NQO1*) were downregulated at 24 h POMx for three oral cancer cell lines (Figure 7A). Moreover, protein expressions for these antioxidant genes are downregulated at 72 h POMx treatment for oral cancer cells (HSC-3 and OC-2) but slightly upregulated for Ca9-22 cells (Figure 7B). These results suggest that mRNA and protein expressions for antioxidant signaling may be differentially regulated between different oral cancer cell lines. Under these differential regulation modes, oxidative stress such as MitoMP depletion and MitoSOX generation were upregulated at 12, 24, and 72 h POMx in three oral cancer cells (Figure 5 and Figure 6). Therefore, antioxidant pathways play a vital function in POMx induced oxidative stress in the present study.

### 4.3. POMx Induces Mitochondrial Impairment in Oral Cancer Cells

In addition to MitoMP and MitoSOX, mitochondrial mass, mtDNA copy number, mtDNA lesion, and mitochondrial biogenesis were also changed after POMx incubation in the present study. Similarly, Resveratrol may mitigate neurotoxicity following Rotenone treatment through promoting mitochondrial mass and DNA copy number [56]. Thus, there is a complex interaction between these mitochondrial functions.

Modulating mitochondrial function is associated with apoptosis. In view of mitochondrial mass change, several treatments may subsequently induce apoptosis. For example, TNFα decreases mitochondrial mass and induces apoptosis in human dermal microvascular endothelial cells (HMEC-1) [57]. In the present study, POMx shows similar results for oral cancer cells. The Mitotracker-detected mitochondrial mass is downregulated at 12 and 24 h POMx treatment but slightly upregulated at 72 h (Figure 8C,D). Similarly, Western blotting shows that mitochondrial resident protein TIM22 is upregulated at 72 h POMx. Accordingly, the mitochondrial mass is dynamically changed over time after POMx treatment of oral cancer cells. The role of POMx-induced mitochondrial mass change warrants a detailed investigation in the future.

mtDNA copy number change may regulate apoptosis. Increasing mtDNA copy number may inhibit apoptosis. In contrast, reducing mtDNA copy number was shown to induce ROS generation and apoptosis in tumor cells [58]. Similarly, 24 and 72 h POMx treatment increased oxidative stress and decreased mtDNA copy number (Figure 9A) in oral cancer cells, leading to apoptosis.

A change of mtDNA damage regulates apoptosis. Single [59] or double [60] strand breaks in mtDNA may induce apoptosis. Moreover, mtDNA damage induces MitoSOX generation and subsequent apoptosis [61]. Similarly, 24 and 72 h POMx treatment causes mtDNA damage (Figure 9B), MitoSOX (Figure 6), and apoptosis (Figure 4). Moreover, oxidative stress also induces oxidative DNA damage of nuclear DNA [62]. Our finding supported this because POMx caused DNA double-strand breaks (γH2AX) in oral cancer cells (Figure 10).

Change of mitochondrial biogenesis change regulates apoptosis. Biogenesis may increase mitochondrial mass and DNA copy number [56] and is associated with apoptosis [63,64]. Similarly, 24 and 72 h POMx treatment inhibits mRNA and protein expressions for mitochondrial biogenesis of gene expression (*TFB2M*, *TFAM*, *POLRMT*, and *TUFM*) in oral cancer cells (Figure 9C,D). This finding supports the notion that a decrease in mitochondrial biogenesis reduces the mitochondrial mass (Figure 8). Moreover, mitochondrial fission factor (MFF) overexpression in breast cancer cells decreases both mitochondrial mass and activity [65]. Since POMx downregulates mitochondrial biogenesis (Figure 9C,D) and mass (Figure 8), it is possible that POMx treatment causes mitochondrial fission and leads to apoptosis of oral cancer cells. It warrants a detailed investigation of the role of mitochondrial fission in POMx treatment for oral cancer cells in the future.

### 4.4. POMx Induces Apoptosis but Inhibits Autophagy in Oral Cancer Cells

POMx and pomegranate leaf extract (PLE) respectively induce apoptosis in human prostate [10] and lung [21] cancer cells. However, no caspase experiments were performed before the present study. Ethanol extracts of pomegranate fruit (PEE) induced apoptosis by cleaving Cas-3 and raising Bax/Bcl-2 ratio in urinary bladder cancer T24 cells [22]. Consistently, 72 h POMx induced apoptosis for oral cancer cells by the results of annexin V expression (Figure 4A) and Western blotting (Figure 4C).

The autophagy pathway is activated to guarantee the elimination of damaged mitochondria to maintain cell survival. In the case where autophagy is reduced, this may lead to cell death without the elimination of damaged mitochondria. This rationale is partly supported by our finding that the increase of apoptosis is accompanied by a decrease in AO-detected autophagy ranging from 12 to 72 h POMx treatment (Figure 4E). These results warrant a detailed investigation of the impact of mitophagy or autophagy upon POMx treatment of oral cancer cells.

## 5. Conclusions

In the present study, the antiproliferation of POMx was evaluated using several types of oral cancer cells, and its detailed mechanisms related to mitochondrial function were explored. POMx treatment shows antiproliferation and apoptosis associated with downregulating antioxidant gene expression and triggering mitochondrial impairment, causing ATP depletion, MitoMP disruption, and MitoSOX generation as well as decreases in mitochondrial mass, mtDNA copy number, and mitochondrial biogenesis. Moreover, both nuclear and mitochondrial DNA damages were induced by POMx incubation in oral cancer cells. In conclusion, POMx provides antiproliferation and apoptosis effects on oral cancer cells through impaired mitochondrial functioning.

## Figures and Tables

**Table 1:** Primer sequences and amplicon lengths for antioxidant- and mitochondrial biogenesis-related genes.

Genes	Forward Primers (5′→3′)	Reverse Primers (5′→3′)	Length
*NFE2L2*	GATCTGCCAACTACTCCCAGGTT [36]	CTGTAACTCAGGAATGGATAATAGCTCC [36]	302 bp
*GCLC*	ACAAGCACCCTCGCTTCAGTACC [36]	CTGCAGGCTTGGAATGTCACCT [36]	232 bp
*TXN*	GAAGCAGATCGAGAGCAAGACTG [36]	GCTCCAGAAAATTCACCCACCT [36]	270 bp
*CAT*	ATGCAGGACAATCAGGGTGGT [36]	CCTCAGTGAAGTTCTTGACCGCT [36]	274 bp
*SOD1*	AGGGCATCATCAATTTCGAGC [37]	CCCAAGTCTCCAACATGCCTC [36]	211 bp
*HMOX1*	CCTTCTTCACCTTCCCCAACAT [36]	GGCAGAATCTTGCACTTTGTTGC [36]	251 bp
*NQO1*	GAAGGACCCTGCGAACTTTCAGTA [36]	GAAAGCACTGCCTTCTTACTCCG [36]	258 bp
*TFB2M*	CTGCTGGAGTGCAATCCAGGTC	TCCAACTACTTTTAAAGGGATGTCTGC	285 bp
*TFAM*	TTAAAGCTCAGAACCCAGATGCA [39]	TTACAGTCTTCAGCTTTTCCTGCG	354 bp
*POLRMT*	CTGAGCGACTTTCCCCAGGAGT	CTTACGTGTGTTGGGCTTTCGG	294 bp
*TUFM*	TGCTCTCTGTGCCCTTGAGGGT	CTTGTGGAACATCTCAATGCCTGTC	277 bp
*GAPDH*	CCTCAACTACATGGTTTACATGTTCC [41]	CAAATGAGCCCCAGCCTTCT [42]	220 bp

**Table 2:** Primer sequences and amplicon lengths for mitochondrial DNA copy number and DNA damage related genes.

Genes	Forward Primers (5′→3′)	Reverse Primers (5′→3′)	Length
*ND1*	CCTCCTACTCCTCATTGTACCCATTC	TGAAGAGTTTTATGGCGTCAGCG	155 bp
*ND1-L*	CCTCCTACTCCTCATTGTACCCATTC	GAGTGTGCCTGCAAAGATGGTAGAG	1203 bp
*ND5*	GTTTCATCCTCGCCTTAGCATGA	AGTCAGGGGTGGAGACCTAATTGG	157 bp
*ND5-L*	GTTTCATCCTCGCCTTAGCATGA	GGTGATGATGGAGGTGGAGATTTG	1190 bp
*GAPDH*	GAAGCTGAGTCATGGGTAGTTGG [44]	GATCTGGTTTCCGGAAGACG [44]	220 bp

*L* indicates the long-run PCR for target genes such as *ND1* and *ND5*.

## Data Availability

Data is contained within the article.

## Gene Accession Numbers

	**Gene**	**Accession number**
RNA	*NFE2L2*	NM_006164.5
	*GCLC*	NM_001498.4
	*TXN*	NM_003329.4
	*CAT*	NM_001752.4
	*SOD1*	NM_000454.4
	*HMOX1*	NM_002133.3
	*NQO1*	NM_000903.3
	*TFB2M*	NM_022366.3
	*TFAM*	NM_003201.3
	*POLRMT*	NM_005035.4
	*TUFM*	NM_003321.5
	*GAPDH*	NM_002046.7
	**Gene**	**Accession number**
DNA	*ND1*	NC_012920.1
	*ND1-L*	
	*ND5*	
	*ND5-L*	
	*GAPDH*	NG_007073.2
